# A Narrative Review of the Metabolic Benefits of GLP-1 and GIP Receptor Agonists in Obesity

**DOI:** 10.3390/healthcare14060734

**Published:** 2026-03-13

**Authors:** Andrew-Hyun Lee, Deborah Jane Holmes-Walker

**Affiliations:** 1 Department of Diabetes and Endocrinology, Westmead Hospital, Cnr Hawkesbury Road and Darcy Road, Westmead, Sydney, NSW 2145, Australia; andrewhyun.lee@health.nsw.gov.au; 2 Sydney Medical School, The University of Sydney, Sydney, NSW 2006, Australia

**Keywords:** glucagon-like peptide-1 receptor agonist, glucose-dependent insulinotropic polypeptide receptor agonist, cardiometabolic outcomes

## Abstract

Glucagon-like peptide-1 (GLP-1) and dual GLP-1 and glucose-dependent insulinotropic polypeptide (GIP) receptor agonists are highly effective therapies for overweight and obesity due to their potent ability to provide significant amounts of weight loss. There is also increasing evidence in their medium- to long-term benefits to metabolic outcomes in those with obesity, due to improvements in weight and glycemia, as well as direct action of GLP-1 on multiple organ systems. This narrative review examines the literature behind the metabolic effects in those with overweight and obesity, as well as the implications of long-term use in regard to the safety and cost-effectiveness of these agents. Improvement and prevention of metabolic disease with GLP-1 therapies remains promising, although studies of longer duration are required to further expand indications and confirm safety of therapy.

## 1. Introduction

The global prevalence of overweight and obesity has continued to rise, with an estimated 890 million adults living with obesity in 2025 [1]. Excess weight is a significant modifiable risk factor for a myriad of metabolic complications, including type 2 diabetes (T2D), atherosclerotic cardiovascular disease, and metabolic-dysfunction-associated steatotic liver disease (MASLD). In particular, visceral fat accumulation and subsequent insulin resistance leads to rises in circulating fatty acids, chronic low-grade inflammation, and adipokine dysregulation, creating a vicious cycle resulting in increased hepatic and pancreatic fat deposition. The interplay of diabetes and excess weight exerts further adverse metabolic effects on cardiovascular risk and liver fibrosis, thereby affecting life expectancy in those with overweight and obesity [2].

It is therefore unsurprising that glucagon-like peptide-1 (GLP-1) receptor agonists and newer dual GLP-1 and glucose-dependent insulinotropic polypeptide (GIP) receptor agonists have rapidly gained prominence due to their favorable effects on excess weight and glycemia. This has been accompanied by an increasing volume of research evaluating their influence on downstream metabolic outcomes in individuals with obesity. Given the rapidly expanding evidence base, a synthesis of current knowledge has become increasingly essential to enable appropriate evaluation of the role of these therapies in obesity care. This narrative review offers a rigorous appraisal of the contemporary evidence on GLP-1- and GIP-based therapies in obesity, with a comparative review of weight loss and diabetes prevention of individual agents from recently published studies. We further highlight the medium- to long-term impacts of therapy to metabolic health, with a focus on cardiovascular outcome trials, MASLD and obstructive sleep apnea (OSA), alongside a discussion of the mechanistic pathways underlying the long-term metabolic benefits and safety outcomes of GLP-1 therapy which are often only briefly addressed in other reviews.

## 2. Materials and Methods

A comprehensive search using PubMed (Medline), EMBASE, and SCOPUS databases was used to retrieve relevant articles published from January 2000 to October 2025 (see Supplementary Material). The literature search was performed in November 2025. Key search terms included “GLP-1 receptor agonists”, “GIP receptor agonists”, “liraglutide”, “semaglutide”, “tirzepatide”, “obesity”, “pre-diabetes”, “weight loss”, “cardiovascular diseases”, “coronary artery disease”, “major adverse cardiovascular events”, “obstructive sleep apnea”, “fatty liver”, and “metabolic dysfunction-associated steatotic liver disease”. Trials assessing systemic complications directly associated with overweight and obesity were reviewed, whilst indirect weight-related outcomes such as chronic kidney disease, osteoarthritis, and dementia were excluded. Randomized placebo-controlled trials were primarily included, and observational studies included when no randomized studies were available. Systematic reviews and meta-analyses were not included, while non-human and non-English studies were also excluded. Only trials in adult participants were included. Titles and abstracts were used for screening, and full texts read for selected articles.

## 3. Results

### 3.1. Weight Loss

The rising popularity in the use of GLP-1 therapies has primarily stemmed from their significant effect on weight reduction, with many of these agents far exceeding the 5% weight loss target that has long been considered clinically meaningful in improving metabolic health [3,4]. Successively higher doses and the development of newer agents have now allowed a percentage weight loss approaching that of metabolic surgery (Table 1).

The dual GLP-1 and GIP analog tirzepatide has demonstrated the greatest reduction in weight, with a 20.9% weight loss achieved with tirzepatide 15 mg weekly at 72 weeks [5]. Tirzepatide commenced in those who had already demonstrated successful weight loss with an intensive lifestyle intervention were able to achieve a further 14.7% weight reduction, for a combined 24.3% weight loss in this select population [6]. Head-to-head studies have shown superiority in weight loss of maximally tolerated tirzepatide to 15 mg (20.2% weight loss) compared to maximally tolerated semaglutide to 2.4 mg (13.2% weight loss) [7]. Semaglutide 7.2 mg weekly has recently revealed weight loss comparable to that of tirzepatide [8], although a head-to-head comparison is unlikely to be performed. Weight nadir occurs at approximately 12–18 months [9], with durability in effect demonstrated out to three years for semaglutide and tirzepatide [10,11]. A slow but gradual weight regain after weight nadir was shown with liraglutide [9].

The degree of weight loss achieved with GLP-1 therapies has been well documented to be blunted by up to one-third in those with T2D. This difference has been demonstrated with all agents, including short-acting GLP-1 therapies such as liraglutide [12,13], as well as the longer acting semaglutide and tirzepatide [14,15] (Table 1). 

**Table 1:** Seminal registry randomized controlled weight loss outcome trials in GLP-1- and GIP-based therapies, in those with and without diabetes.

Medication	Study	Trial Characteristics	Demographics	Intervention	Comparator	Weight Loss Outcomes
Liraglutide	SCALE Obesity and Prediabetes [12]	Multicenter, randomized, prospective, double-blinded, 56 weeks N = 3731	BMI ≥ 30 kg/m^2^ or ≥27 kg/m^2^ with dyslipidaemia or hypertension, without diabetes	Liraglutide 3 mg sc daily	Placebo	Liraglutide 3 mg: 8.0% Placebo: 2.6%
	SCALE Diabetes [13]	Multicenter, randomized, prospective, double-blinded, 56 weeks N = 396	BMI ≥ 27 kg/m^2^, T2D with HbA1c 7–10% (53–86 mmol/mol)	Liraglutide 1.8 mg or 3 mg sc daily	Placebo	Liraglutide 1.8 mg: 4.7% Liraglutide 3 mg: 6.0% Placebo: 2.0%
Semaglutide (subcutaneous)	STEP 1 [16]	Multicenter, randomized, prospective, double-blinded, 68 weeks N = 1961	BMI ≥ 30 kg/m^2^ or ≥27 kg/m^2^ with one or more weight-related complications, excluding diabetes	Semaglutide 2.4 mg sc once weekly	Placebo	Semaglutide 2.4 mg: 14.9% Placebo: 2.4%
	STEP 2 [17]	Multicenter, randomized, prospective, double-blinded, 68 weeks N = 1210	BMI ≥ 27 kg/m^2^, T2D with HbA1c 7–10% (53–86 mmol/mol)	Semaglutide 1 mg or 2.4 mg sc once weekly	Placebo	Semaglutide 1 mg: 7.0% Semaglutide 2.4 mg: 9.6% Placebo: 2.4%
	STEP UP [8]	Multicenter, randomized, prospective, double-blinded, 72 weeks N = 1407	BMI ≥ 30 kg/m^2^, without diabetes	Semaglutide 2.4 mg or 7.2 mg sc weekly	Placebo	Semaglutide 2.4 mg: 15.6% Semaglutide 7.2 mg: 18.7% Placebo: 3.9%
	STEP UP T2D [18]	Multicenter, randomized, prospective, double-blinded, 72 weeks N = 512	BMI ≥ 30 kg/m^2^, T2D with HbA1c 7–10% (53–86 mmol/mol)	Semaglutide 2.4 mg or 7.2 mg sc weekly	Placebo	Semaglutide 2.4 mg: 10.4% Semaglutide 7.2 mg: 13.2% Placebo: 3.9%
Semaglutide (oral)	OASIS 1 [19]	Multicenter, randomized, prospective, double-blinded, 68 weeks N = 709	BMI ≥ 30 kg/m^2^ or ≥27 kg/m^2^ with one or more weight-related complications, excluding diabetes	Semaglutide 50 mg oral daily	Placebo	Semaglutide 50 mg: 15.1% Placebo: 2.4%
	OASIS 4 [20]	Multicenter, randomized, prospective, double-blinded, 64 weeks N = 205	BMI ≥ 30 kg/m^2^ or ≥27 kg/m^2^ with one or more weight-related complications, excluding diabetes	Semaglutide 25 mg oral daily	Placebo	Semaglutide 25 mg: 13.6% Placebo: 2.2%
Tirzepatide	SURMOUNT-1 [5]	Multicenter, randomized, prospective, double-blinded, 72 weeks N = 2.539	BMI ≥ 30 kg/m^2^ or ≥27 kg/m^2^ with one or more weight-related complications, excluding diabetes	Tirzepatide 5 mg, 10 mg or 15 mg sc once weekly	Placebo	Tirzepatide 5 mg: 15.0% Tirzepatide 10 mg: 19.5% Tirzepatide 15 mg: 20.9% Placebo: 3.1%
	SURMOUNT-2 [15]	Multicenter, randomized, prospective, double-blinded, 72 weeks N = 1514	BMI ≥ 27 kg/m^2^, T2D with HbA1c 7–10% (53–86 mmol/mol)	Tirzepatide 10 mg or 15 mg sc once weekly	Placebo	Tirzepatide 10 mg: 12.8% Tirzepatide 15 mg: 14.7% Placebo: 3.2%

BMI = body mass index; HbA1c = glycosylated hemoglobin; sc = subcutaneous; T2D = type 2 diabetes.

### 3.2. Diabetes Prevention

Obesity is a key risk factor in the development of T2D, and GLP-1-based therapies have the potential to play a crucial role in curbing the accelerating incidence of diabetes (Table 2). A 3-year trial of liraglutide in those with pre-diabetes and obesity demonstrated reductions in diabetes diagnosis by 70% and increased reversion to normoglycemia by 80% compared to placebo [9]. A smaller study in women with previous gestational diabetes reduced the prevalence of pre-diabetes by half with liraglutide [21]. Reductions in T2D diagnosis have also been observed with semaglutide with an 80% reduction in incident diabetes in a comorbid population [22], and similarly with tirzepatide with a reduction in incident diabetes by 90% [11].

The degree of diabetes risk prevention is greater than established in lifestyle intervention trials (Diabetes Prevention Program [23], Finnish Diabetes Prevention Study [24]), which demonstrated a 55–60% reduction in incident diabetes after 3 years. A 31% risk reduction was observed with metformin compared to placebo [23], while reductions in annualized diabetes incidence ranging between 70 and 80% have been noted with phentermine/topiramate [25] and pioglitazone [26] after 2 years.

The durability of GLP-1 therapy response for maintaining normoglycemia may vary depending on the agent, with the HbA1c nadir noted at 20 weeks for semaglutide in those with pre-diabetes and obesity, followed by a slight parallel increase in HbA1c over time in both treatment and placebo arms [22]. Conversely, tirzepatide may confer a more durable response [11], although direct comparisons are difficult.

**Table 2:** Randomized controlled diabetes prevention trials in GLP-1- and GIP-based therapies.

Medication	Study	Trial Characteristics	Demographics	Intervention	Comparator	Glycemic Outcomes
Liraglutide	[9]	Multicenter, randomized, prospective, double-blinded, 160 weeks followed by 12 weeks cessation N = 2254	Pre-diabetes, BMI ≥ 30 kg/m^2^ or ≥27 kg/m^2^ with co-morbidities	Liraglutide 3 mg sc daily	Placebo	T2D diagnosis Week 160: 1.8% (liraglutide 3 g), 6.2% (placebo) Week 172: 2.1% (liraglutide), 6.4% (placebo) Reversion to normoglycemia Week 160: 66% (liraglutide), 36% (placebo) Week 172: 50% (liraglutide), 36% (placebo)
	[21]	Single center (Denmark), randomized, prospective, double-blinded, 52 weeks N = 104	Previous gestational diabetes requiring metformin or insulin, BMI ≥ 30 kg/m^2^, delivery ≤ 25 months ago	Liraglutide 1.8 mg sc daily	Placebo	T2D diagnosis Week 52: 3% (liraglutide), 8% (placebo) * Pre-diabetes diagnosis Week 52: 27% (liraglutide), 58% (placebo)
Semaglutide	STEP 10 [27]	Multicenter, randomized, prospective, double-blinded, 52 weeks followed by 28 weeks cessation N = 138	Pre-diabetes, BMI ≥ 30 kg/m^2^	Semaglutide 2.4 mg sc weekly	Placebo	T2D diagnosis Week 52: 1% (semaglutide), 2% (placebo) ** Week 80: 3% (semaglutide), 5% (placebo) ** Reversion to normoglycemia Week 52: 81% (semaglutide), 14% (placebo) Week 80: 44% (semaglutide), 18% (placebo) **
	Substudy of SELECT [22]	Multicenter, randomized, prospective, double-blinded, 156 weeks N = 17,604	Age ≥ 45, BMI ≥ 27 kg/m^2^, pre-existing cardiovascular disease, without diabetes	Semaglutide 2.4 mg sc weekly	Placebo	T2D diagnosis Week 156: 1.5% (semaglutide), 6.9% (placebo) Reversion to normoglycemia Week 156: 69.5% (semaglutide), 35.8% (placebo)
Tirzepatide	Substudy of SURMOUNT-1 [11]	Multicenter, randomized, prospective, double-blinded, 176 weeks, followed by 17 weeks off treatment N = 2539	Pre-diabetes, BMI ≥ 30 kg/m^2^ or ≥27 kg/m^2^ with co-morbidities	Tirzepatide 5 mg, 10 mg, or 15 mg sc weekly	Placebo	T2D diagnosis Week 176: 1.3% (tirzepatide), 13.3% (placebo) Week 193: 2.4% (tirzepatide), 13.7% (placebo)

BMI = body mass index; sc = subcutaneous. * Did not reach statistical significance. ** Exploratory analyses, no statistical analysis completed.

### 3.3. Cardiovascular Effects

Obesity is a significant risk factor for the development of cardiovascular disease and is associated with increased cardiovascular mortality [28]. A reduction in cardiovascular morbidity and mortality remains one of the principal long-term goals of weight management; however, the benefits of weight loss itself on cardiovascular outcomes are mixed [29].

Cardiovascular benefits have been observed with the majority of GLP-1 therapies (barring lixisenatide and once weekly exenatide [30,31]) in the T2D trials. Significant reductions in major adverse cardiovascular events (MACE) have been demonstrated with liraglutide (13%) [32], semaglutide (26%) [33], and dulaglutide (10%) [34] (Table 3), although the low representation of cardiovascular disease in the dulaglutide REWIND trial may explain its smaller effect on MACE (31%, compared to >80% in the other trials). Reductions in MACE with semaglutide and dulaglutide over 2 and 5 years respectively were driven by decreases in non-fatal stroke, while no significant reductions in non-fatal myocardial infarction or cardiovascular death were observed compared to placebo. Initial 1-year oral semaglutide trials had not demonstrated superiority compared to placebo in MACE [35], although a trial of longer duration over 4 years in those with higher cardiovascular risk observed a 14% reduction in MACE [36].

Cardiovascular outcome trials in those with obesity without comorbid diabetes are lacking, with the SELECT trial for semaglutide being the only current randomized controlled trial in this population. Use of semaglutide 2.4 mg revealed a 20% reduction in MACE over a mean follow up of 40 months [37]. A combined post hoc analysis of several liraglutide SCALE trials in those without diabetes revealed a non-inferiority of liraglutide to placebo in regard to cardiovascular outcomes, although these trials were limited by duration to 56 weeks [38].

Previous retrospective cohort studies of GLP-1 therapies in T2D suggested superiority of tirzepatide in cardiovascular outcomes [39]. However, recent results from the SURPASS-CVOT randomized trial of tirzepatide 15 mg and dulaglutide 1.5 mg with a median 4-year duration of follow-up showed non-inferiority of tirzepatide over dulaglutide in MACE [40]. This occurred despite superiority of tirzepatide over dulaglutide in weight loss and improvement in HbA1c, suggesting a common mechanism beyond glycemia that mediates the cardiovascular effects of GLP-1 therapy. A cardiovascular outcome trial for tirzepatide in those with obesity but without diabetes is pending [41].

**Table 3:** Randomized controlled cardiovascular outcome trials in GLP-1- and GIP-based therapies, in those with and without diabetes.

Medication	Study	Trial Characteristics	Demographics	Intervention	Comparator	Cardiovascular Outcomes
Liraglutide	LEADER [32]	Multicenter, randomized, prospective, double-blinded, 42–60 months (median 3.8 years) N = 9340	T2D, high CV risk	Liraglutide 1.8 mg sc daily	Placebo	MACE: 13.0% (liraglutide), 14.9% (placebo) HR 0.87 (95%CI 0.78–0.97)
Semaglutide (subcutaneous)	SUSTAIN-6 [33]	Multicenter, randomized, prospective, double-blinded, 104 weeks N = 3927	Age ≥ 50, T2D with HbA1c ≥ 7% (53 mmol/mol), established CV disease or chronic kidney disease	Semaglutide 0.5 mg or 1 mg sc weekly	Placebo	MACE: 6.6% (semaglutide), 8.9% (placebo) HR 0.74 (95%CI 0.58–0.95)
	SELECT [37]	Multicenter, randomized, prospective, double-blinded, mean follow-up 39.8 months N = 17,604	Age ≥ 45, BMI ≥ 27 kg/m^2^, established CV disease, without diabetes	Semaglutide 2.4 mg sc weekly	Placebo	MACE: 6.5% (semaglutide), 8.0% (placebo) HR 0.80 (95%CI 0.72–0.90)
Semaglutide (oral)	PIONEER 6 [35]	Multicenter, randomized, prospective, double-blinded, median follow-up 15.9 months N = 3183	Age ≥ 50, T2D, high CV risk	Semaglutide 14 mg oral daily	Placebo	MACE: 3.8% (semaglutide), 4.8 (placebo) HR 0.79 (95%CI 0.57–1.11)
	SOUL [36]	Multicenter, randomized, prospective, double-blinded, median follow-up 49.5 months N = 9650	Age ≥ 50, T2D with HbA1c 6.5–10% (48–86 mmol/mol), established CV disease or chronic kidney disease	Semaglutide 14 mg oral daily	Placebo	MACE: 12.0% (semaglutide), 13.8% (placebo) HR 0.86 (95%CI 0.77–0.96)
Dulaglutide	REWIND [34]	Multicenter, randomized, prospective, double-blinded, median follow-up 5.4 years N = 9901	Age ≥ 50, T2D with HbA1c ≤ 9.5% (80 mmol/mol), high CV risk	Dulaglutide 1.5 mg sc weekly	Placebo	MACE: 12.0% (dulaglutide), 13.4% (placebo) HR 0.88 (95%CI 0.79–0.99)
Tirzepatide	SURPASS-CVOT [40]	Multicenter, randomized, prospective, double-blinded, median follow-up 4.0 years N = 13,299	Age ≥ 40, T2D with HbA1c 7.0–10.5% (53–91 mmol/mol), BMI ≥ 25 kg/m^2^, established CV disease	Tirzepatide 15 mg sc weekly	Dulaglutide 1.5 mg sc weekly	MACE: 12.2% (tirzepatide), 13.1% (dulaglutide) HR 0.92 (95%CI 0.83–1.01)

BMI = body mass index; HbA1c = glycosylated hemoglobin; T2D = type 2 diabetes; CV = cardiovascular; sc = subcutaneous; MACE = major adverse cardiovascular events; HR = hazard ratio; CI = confidence interval.

### 3.4. Hepatic Effects

MASLD encompasses a spectrum of chronic liver conditions, including isolated steatosis, steatohepatitis (MASH; defined by inflammation and cellular injury), with varying degrees of fibrosis that can result in cirrhosis. Obesity and subsequent insulin resistance are key drivers in the development of MASLD, with an insulin-resistant environment resulting in increased peripheral lipolysis and free fatty acid delivery to the liver, with increased glucose-dependent de novo hepatic lipogenesis [42].

Although GLP-1 receptors are not thought to be expressed in hepatocytes, their pleiotropic effects on weight, glucose utilization and insulin sensitivity have been shown to be beneficial in MASLD. Various liraglutide trials have demonstrated reduction in liver fat content by 19–32% [43], alongside a resolution in steatohepatitis in 39% (compared to 9% in placebo) [44]. The recent interim analysis for the ESSENCE trial in semaglutide 2.4 mg in those with biopsy-proven MASH with stage 2–3 fibrosis revealed resolution of MASH without progression of fibrosis in 63% (34% in placebo) at 72 weeks. An improvement in fibrosis without a worsening of MASH was observed in 37% (22% in placebo) although this result did not meet statistical significance. Outcomes were no different in those with T2D compared to those without, with 56% of the cohort exhibiting T2D [45].

Similarly, the SYNERGY-NASH trial of tirzepatide in those with MASH with stage 2–3 fibrosis demonstrated resolution in MASH in 62% at the 15 mg dose (10% in placebo) and improvements in fibrosis in 51% (30% in placebo) at 52 weeks, with the latter not meeting statistical significance. A dose–response relationship was observed, with lower doses of tirzepatide exhibiting lower rates of MASH resolution. T2D represented 58% of the study population, although no subgroup analysis was provided [46].

Early treatment with GLP-1 therapies remains time-critical, as improvements in fibrosis or steatohepatitis have not been observed in those with established cirrhosis [47,48], although a population-based cohort study has suggested reductions in decompensated cirrhosis and hepatic encephalopathy with GLP-1 therapy use in cirrhosis [49]. The effects of these agents in early steatotic liver disease remain unexplored. The planned 240-week trial of semaglutide will provide a further understanding of the long-term benefits [45].

### 3.5. Respiratory Effects

The risk of OSA increases six-fold for every 10% increase in body weight, with an increased propensity for airway collapse from excess adipose around the pharyngeal airways. Alterations in upper airway muscle function and lung volumes in obesity can further exacerbate airway instability [50].

Tirzepatide is the first medical therapy approved for OSA by the Food and Drug Administration, with the SURMOUNT-OSA trial demonstrating improvements in the apnea-hypopnea index (AHI) at 52 weeks in those with obesity and moderate–severe OSA [51]. The mean AHI improved by greater than half, with a reduction by 29.3 events/h (placebo −5.5 events/h) in those using positive airway pressure (PAP) and a reduction of 25.3 events/h (placebo −5.3 events/h) in those not using PAP therapy, which meets clinically meaningful thresholds [52]. Improvements in hypoxic burden by 60–70% min/h with tirzepatide may correlate with a decreased cardiovascular risk, although long-term data remain pending. Sleep-related patient-reported outcomes also significantly improved with tirzepatide.

Randomized controlled trials with liraglutide in moderate–severe OSA have also shown reductions in the AHI and hypoxia in those with and without PAP therapy, although improvements in the AHI have been modest at 16–25% [53,54,55]. One study revealed reductions in cardiovascular risk indicators (such as coronary plaque volume) with PAP or concurrent PAP and liraglutide treatment, but not with liraglutide alone, although this study was limited by its short duration [55]. In contrast, the SURMOUNT-OSA trial demonstrated reductions in blood pressure and C-reactive protein with tirzepatide, and the SURMOUNT-Morbidity and Mortality in Obesity trial will likely provide additional information on long-term cardiovascular outcomes.

## 4. Discussion

GLP-1- and GIP-based therapies constitute a significant evolution in obesity management, with degrees of weight loss rivaling those observed with metabolic surgery. This weight loss is predominantly achieved through reductions in caloric intake due to a GLP-1–mediated suppression of appetite in the central modulation of hunger [56]. Activation of GIP receptors centrally may also explain the larger reductions in weight observed with tirzepatide [57]. The greater weight loss potential of GLP-1 therapy carries significant clinical relevance, given the well-established association between escalating weight-loss thresholds and corresponding improvements or remission of obesity-related complications [3,4]. For instance, the ability to achieve a weight loss greater than 10–15%, previously only readily attainable through metabolic surgery, is likely to account for the benefits for MASLD and OSA outcomes [4].

Weight loss achieved with GLP-1- and GIP-based therapies has, however, consistently been demonstrated to be blunted in individuals with concurrent obesity and diabetes. This phenomenon was first observed with liraglutide [12,13] and has been more pronounced with the longer-acting GLP-1 therapies [15,17]. This attenuation may in fact be underestimated in diabetes trials due to exclusion of those with more severe hyperglycemia. Greater diabetes severity, as assessed by higher HbA1c, insulin use, number of diabetes medications, and duration of diabetes, has been associated with a stepwise reduction in weight loss with these agents [58]. Reasons for reduced weight loss efficacy in those with T2D are unclear, although improvements in glycemia that occur in diabetes resulting in reduced glycosuria may account for a plateau in its weight benefit. Use of insulin and sulfonylureas, which contribute to weight gain, may also contribute; however, insulin use was not highly represented in many of the trials in T2D. Trial populations in diabetes studies also tend to be older and leaner, and may therefore have less excess weight to lose. A greater reduction in energy expenditure has been observed in T2D, which may also lead to a reduced effect on weight loss in those with diabetes [59].

Although weight loss remains central to many of the metabolic effects of GLP-1 therapy, weight loss alone does not fully account for the breadth of metabolic improvements observed. The insulinotropic actions of GLP-1 and GIP certainly contribute to several metabolic benefits, including diabetes remission and improvements in MASLD. Notably, the T2D prevention trials revealed that the magnitude of weight loss and baseline glycemia (a marker of residual β- cell function) both independently predicted regression of hyperglycemia, highlighting the combined influence of enhanced insulin sensitivity from weight loss and augmented β-cell function via the incretin effect [11,22]. Post hoc analyses suggested that 25% of semaglutide’s and 40–55% of tirzepatide’s effects on reducing the risk of T2D could be accounted for by weight loss [11,22]. Similarly, there is both preclinical [60] and clinical evidence [61] to suggest that at least some of the improvements in MASLD outcomes are weight loss-independent. Direct GLP-1 receptor activation on liver adipose tissue has been associated with reduced inflammation and oxidative stress, which is critical in the progression of MASLD to fibrosis and cirrhosis [62,63]. Increased adiponectin secretion due to GLP-1 action may also improve insulin sensitivity directly [64].

The widespread expression of GLP-1 receptors may also explain the other pleiotropic actions of GLP-1 therapy. For example, there is now emerging evidence for GLP-1 receptor expression on cardiomyocytes and vascular endothelial cells [65,66,67], which may provide an explanation for the cardioprotective benefits of GLP-1 therapy which have been shown to be independent of baseline adiposity and weight loss [68]. Receptor activation in cardiomyocytes has been shown to increase glucose uptake and reduce oxidative stress and apoptosis, while vasodilation occurs due to GLP-1 action on the endothelium (including coronary blood vessels) [65,66,67]. Direct GLP-1 effects on the kidney via the renin–angiotensin–aldosterone system also further modulate vascular tone, resulting in a reduction in blood pressure [69,70]. Similarly, activation of the GIP pathway may be involved in suppressing monocyte and macrophage activation in atherosclerotic plaques [71] and in the suppression of cardiomyocyte enlargement, apoptosis, and interstitial fibrosis [72]. GLP-1 therapies appear to only have a small effect on low-density lipoprotein cholesterol which is insufficient to account for the reduction in MACE [73].

Despite the encouraging short- to medium-term data for GLP-1 therapy, the long-term impacts of therapy on metabolic outcomes remain uncertain in the absence of extended longitudinal data. Continued therapy is, however, essential for the sustained management of weight excess as significant weight regain has been observed on treatment cessation. A mean 2.2 kg weight regain was observed after cessation of liraglutide [74], while the STEP 1 extension trial of semaglutide 2.4 mg found two-thirds of the weight lost had been regained after a year (from −17.3% at treatment discontinuation to −5.6% at 1 year) [75]. Similarly, a 55% weight regain occurred at 1 year after cessation of tirzepatide 10–15 mg (from −20.9% at treatment discontinuation to −9.5%) [76], although participants had not reached weight nadir at time of cessation unlike in the semaglutide trial. The weight regains did not appear to plateau at trial end for either semaglutide or tirzepatide. Rebound in other metabolic parameters such as waist circumference, systolic blood pressure, and glycemia have also been observed on discontinuation of GLP-1 therapy, although longer term longitudinal studies are required to characterize cardiometabolic risk after treatment cessation [77].

Continued use is, however, often limited by gastrointestinal intolerances which are common side effects of GLP-1 therapy. These occur due to a combination of slowed gastric emptying and activation of central nervous aversive responses [78]. One-half to two-thirds of treatment discontinuation occur due to gastrointestinal side effects [12,15,17,18] with discontinuation rates ranging from 7 to 10% with liraglutide [12,13] and 5–7% with semaglutide [8,16,17,18] and tirzepatide [5,15]. Short-acting GLP-1 therapies have been associated with a higher incidence of nausea and vomiting [79], potentially secondary to higher penetrance across the blood–brain barrier [80] or abrupt changes in gastrointestinal motility due to faster metabolic clearance [79]. Long-acting GLP-1 therapies, in particular semaglutide, have, however, been associated with a higher incidence of diarrhea, possibly secondary to sustained GLP-1 receptor activation leading to enhanced intestinal motility [79]. GIP agonism has been hypothesized to reduce gastrointestinal intolerances with tirzepatide, possibly due to its central actions in the hypothalamus and brainstem [81]. Anti-emetic properties have been observed in pre-clinical [82] and clinical data [81] with GIP agonism, although diarrhea has been observed to be highest with tirzepatide as compared with other GLP-1 therapies [79].

GLP-1 therapies have been associated with a range of other adverse outcomes, and the scope of the following discussion will focus on those related to weight loss. Gallbladder adverse events, particularly cholelithiasis and cholecystitis, are known complications from weight loss due to changes in the lithogenicity of bile and gallbladder stasis [83]. Gallbladder events were increased by 30–35% in meta-analyses of GLP-1 therapies [84,85,86,87]. The degree of weight loss as well as a higher treatment dose and duration have been associated with increased risk of cholelithiasis [84,85], although persistently elevated risk despite adjustment for weight loss suggests that the cause may not be fully explained by a rapid loss in weight [86]. Direct effects of GLP-1 receptor activation on the biliary system have been postulated as a cause for biliary stasis [88].

The substantial weight loss achieved with GLP-1 therapies has also raised concerns about concurrent reductions in muscle mass, which may in turn contribute to plateauing of weight through reductions in basal metabolic rate. Although such reductions in muscle mass are anticipated and comparable to those observed with dietary or surgical weight loss interventions [89], the potential for sarcopenia warrants careful consideration. Lean mass, often used as a surrogate for muscle mass, has been reported in a number of registry trials. Semaglutide 2.4 mg was associated with the largest loss in lean mass at 13.2%, which represented 45.2% of the total weight lost [16]. Despite this, a sub-analysis of STEP 1 revealed a favorable increase in lean mass relative to total body or fat mass [90]. Similarly, although tirzepatide exhibited reductions in lean body mass by 10.9% (representing 25.7% of the total weight lost) [5], the increase in the ratio of lean mass to fat mass was greater in those on tirzepatide compared to placebo [91].

Data from these trials [5,16,90,91], however, needs to be taken with caution as a more rigorous diet and exercise guidance may have minimized muscle loss. In addition, lean mass (a sum of all non-adipose components), remains debated as a useful surrogate for muscle mass, particularly with trials opting to use the less accurate dual energy X-ray absorptiometry (DXA) over magnetic resonance imaging (MRI) [89]. Instead, muscle quality, which can be influenced by the presence of myosteatosis and muscle fiber composition, may provide a composite picture. The SURPASS-3-MRI trial demonstrated greater than predicted reductions in muscle fat infiltration with tirzepatide, alongside as-predicted decreases in muscle volume [92,93]. Liraglutide displayed similar improvements in muscle fat infiltration [94,95].

Adequate protein intake and resistance training are often suggested as strategies to mitigate muscle loss, although there are no randomized controlled data for GLP-1 therapies [95,96]. Resistance training can minimize lean mass loss in adults with obesity (in the absence of GLP-1 therapy) [97], including in studies of elderly with sarcopenic obesity, which demonstrated decreases in body fat by 1.5% and increases in muscle mass by 2.7% with resistance training [98]. Overall protein intake of 1–1.2 g/kg/day in older adults and 1.2–1.5 g/kg/day in those with chronic diseases has also been recommended from sarcopenic obesity trials [99]. Leucine along with other micronutrient supplements as well as microbiome manipulation has also been suggested to prevent muscle loss in the elderly, although heterogeneity in the trial design limits the ability to make clinical recommendations [100].

Body composition data after cessation of GLP-1 therapy is an area requiring further research, particularly in the context of significant weight rebound observed after cessation of GLP-1 therapy. Disproportionate fat regain has been observed in other weight loss settings, with weight cycling potentially predisposing individuals to sarcopenic obesity [101]. Considering the high discontinuation and re-initiation rates in users of GLP-1 therapies [102], this is a significant concern.

Long-term maintenance of GLP-1 therapy relies on two factors; one is to minimize side effects and the second is to improve affordability and supply issues to ensure continued use. Despite the benefits of GLP-1 therapy, the cost has remained a significant barrier for access to therapy, impacting those from lower socioeconomic backgrounds with higher obesity rates and comorbidities who would potentially derive greater benefit. Various cost-effectiveness analyses have called into question their high cost across different health care systems [103,104,105]. As a consequence of their high cost, GLP-1 therapy has been shown to be less cost-effective than metabolic surgery [106] and other anti-obesity medications [103,107], yet their utility and accessibility as compared with metabolic surgery should mean they are used as first-line treatment for the management of obesity. Semaglutide demonstrated the highest cost-effectiveness amongst the GLP-1 therapies [103,104,105]. A full cost–benefit analysis is missing as there is currently no longer-term data of use beyond 72 weeks, with existing modeling relying on short-term clinical trial effects projected over a lifetime.

## 5. Conclusions

The increasing burden of obesity poses significant health, economic, and societal challenges, and GLP-1- and GIP-based therapies can provide a pivotal shift in managing overweight and obesity. Their effects outside of weight loss and glycemic benefit, including cardiovascular, hepatic, and sleep outcomes, show significant promise in improving the survival and quality of life in the millions of individuals affected by these metabolic conditions. Longer term data evaluating the effects of GLP-1 therapy (as well as the effects of treatment cessation) on metabolic outcomes and body composition are warranted. Efficacy and safety in diverse populations, including in the geriatric age group with sarcopenic obesity and in those with chronic end stage kidney disease, are also required. Finally, the major barrier in health care remains equity of access to medication, with the cost burden preventing use of medication in those at greatest risk of obesity and diabetes. Studies evaluating the longer term cost benefit of GLP-1 and GIP therapies through prevention of metabolic disease are required to advocate for greater equity of access.

## Data Availability

Data sharing is not applicable. No new data were created or analyzed in this study.

## Supplementary Materials

The following supporting information can be downloaded at: https://www.mdpi.com/article/10.3390/healthcare14060734/s1, Supplementary S1. Search terms. Supplementary S2. PRISMA flow diagram. Ref. [108] is cited in the supplementary materials file.

## Abbreviations

The following abbreviations are used in this manuscript:

GLP-1	Glucagon-like peptide-1 (GLP-1)
GIP	Glucose-dependent insulinotropic polypeptide
T2D	Type 2 diabetes
CV	Cardiovascular
MACE	Major adverse cardiovascular event
MASLD	Metabolic dysfunction-associated steatotic liver disease
MASH	Metabolic dysfunction-associated steatohepatitis
OSA	Obstructive sleep apnea
AHI	Apnea-hypopnea index
PAP	Positive airway pressure
BMI	Body mass index
DXA	Dual energy X-ray absorptiometry
MRI	Magnetic resonance imaging
SC	Subcutaneous
HR	Hazard ratio
CI	Confidence interval
