# Supplementary material for: A Narrative Review of the Metabolic Benefits of GLP-1 and GIP Receptor Agonists in Obesity
Source: Healthcare (Basel). 2026 Mar 13;14(6):734. doi: 10.3390/healthcare14060734 (PMC13026324; doi:10.3390/healthcare14060734)
Supplement: Supplementary file 1 [file healthcare-14-00734-s001.zip › healthcare-4142756-supplementary.pdf]

Supplementary S1. Search terms

- Glucagon-Like Peptide-1 Receptor Agonists OR GIP receptor agonist OR tirzepatide OR semaglutide OR liraglutide OR dulaglutide
  AND
  Obesity
  AND
  Weight loss/Body weight loss
- Glucagon-Like Peptide-1 Receptor Agonists OR GIP receptor agonist OR tirzepatide OR semaglutide OR liraglutide OR dulaglutide
  AND
  Obesity
  AND
  Diabetes OR prediabetic state/Impaired glucose tolerance
- Glucagon-Like Peptide-1 Receptor Agonists OR GIP receptor agonist OR tirzepatide OR semaglutide OR liraglutide OR dulaglutide
  AND
  Obesity
  Cardiovascular diseases OR myocardial ischemia/heart muscle ischemia OR ischemic stroke OR stroke/cerebrovascular accident OR Coronary Artery Disease OR major adverse cardiovascular events
- Glucagon-Like Peptide-1 Receptor Agonists OR GIP receptor agonist OR tirzepatide OR semaglutide OR liraglutide OR dulaglutide
  AND
  Obesity
  Fatty Liver OR Non-alcoholic Fatty Liver Disease OR Metabolic Dysfunction-Associated Steatotic Liver Disease
- Glucagon-Like Peptide-1 Receptor Agonists OR GIP receptor agonist OR tirzepatide OR semaglutide OR liraglutide OR dulaglutide
  AND
  Obesity
  AND
  Sleep Apnea, Obstructive

Search limited to years January 2000 – October 2025; and randomized controlled trials and clinical trial phase III, IV trials (when filter available). Systematic reviews and meta-analyses removed. Observational studies only included when no randomized controlled trial data available.

Supplementary S2. PRISMA flow diagram

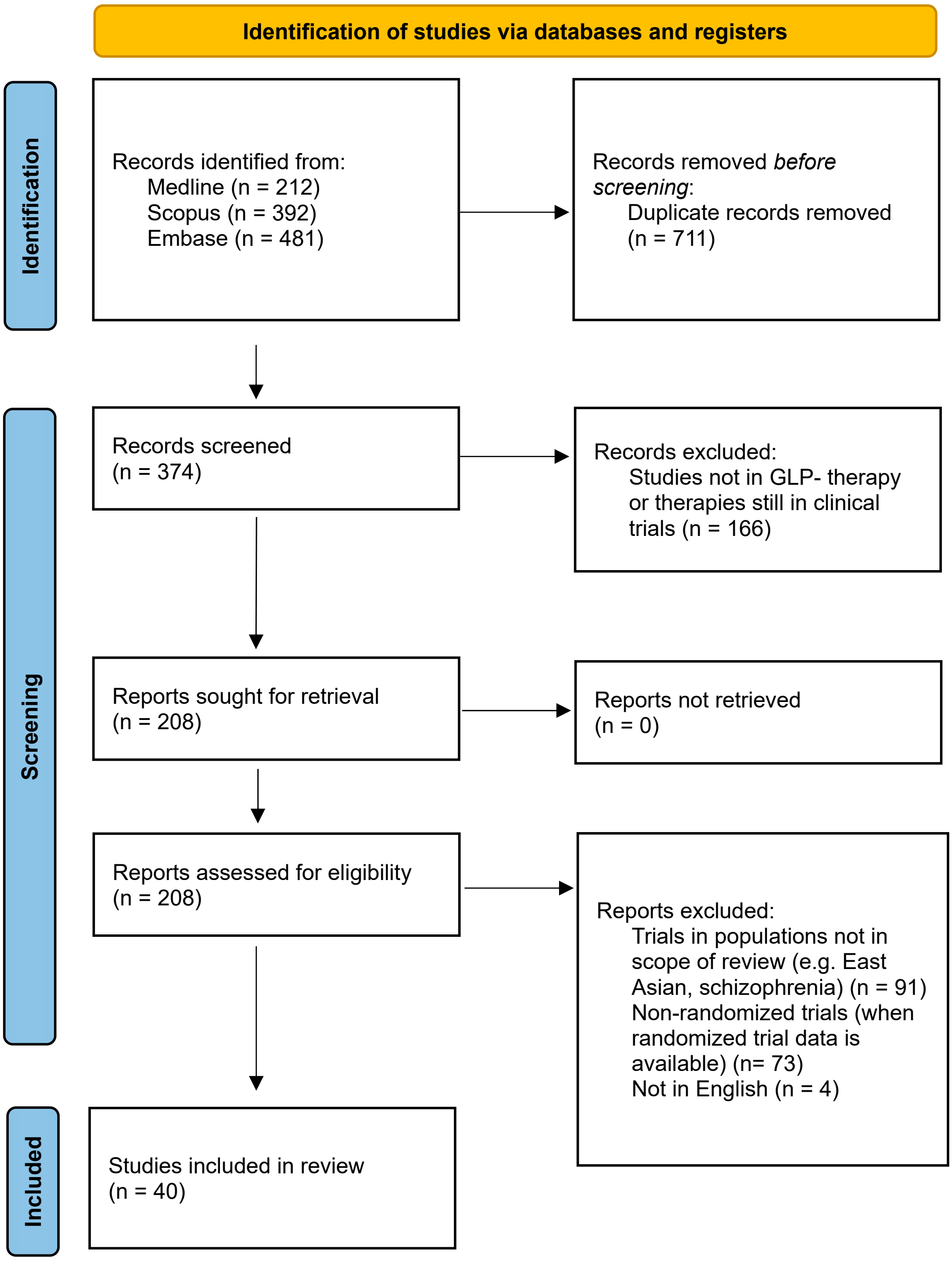

Source: Page MJ, et al. BMJ 2021;372:n71. doi: 10.1136/bmj.n71.

This work is licensed under CC BY 4.0. To view a copy of this license, visit https://creativecommons.org/licenses/by/4.0/
